# Adjuvant hysterectomy for treatment of residual disease in patients with cervical cancer treated with radiation therapy

**DOI:** 10.1038/sj.bjc.6604619

**Published:** 2008-10-14

**Authors:** T Ota, N Takeshima, T Tabata, K Hasumi, K Takizawa

**Affiliations:** 1 Department of Gynecology, Cancer Institute Hospital, Ariake 3-10-6, Koutou-ku, Tokyo 135-8550, Japan

**Keywords:** cervical carcinoma, radiation, residual disease, hysterectomy

## Abstract

The objective of this retrospective study was to determine the efficacy of adjuvant hysterectomy for treatment of residual disease in cervical carcinoma treated with radiation therapy. Between 1971 and 1996, 1590 patients with carcinoma of the uterine cervix (stages I–IIIb) were treated with radiation therapy. Three months after completion of radiation therapy, the status of local control was investigated, and total abdominal hysterectomy was performed in cases in which central residual disease existed in the cervix. Of the 1590 patients, residual disease was identified in 162 patients. Among these patients, 35 showed an absence of distant metastasis or lateral parametrial invasion and underwent hysterectomy. The overall 5- and 10-year survival rates for these patients were 68.6 and 65.7%, respectively. There was no significant difference in survival between patients with squamous cell carcinoma and those with non-squamous cell carcinoma or between patients with stage I/II carcinoma and those with stage III carcinoma. With respect to treatment-related morbidity, five (14.3%) patients suffered grade III or IV complications after hysterectomy. Adjuvant hysterectomy is an effective addition to radiation therapy in the treatment of cervical cancer, even in patients with stage III disease and in those with non-squamous cell carcinoma.

Cervical cancer is one of the most common cancers in women worldwide. Annually, it is estimated that 493 000 women worldwide will be diagnosed with cervical carcinoma and that 273 000 will die from the disease (Parkin *et al*, 2005). Traditionally, radical hysterectomy or radiation therapy alone has been accepted as standard treatment for early-stage invasive cervical cancer, and locally advanced cancer has been treated by radiation therapy alone, consisting of a combination of high-dose-rate intracavitary brachytherapy (ICBT) and external beam radiation therapy (EBRT) (Coia *et al*, 1990; Komaki *et al*, 1995; Barillot *et al*, 1997). In the last few years, substantial advances in the management of locally advanced cervical cancer have been reported. Five randomised trials have shown improved survival and local control when cisplatin-based chemotherapy is performed concurrently with radiation therapy in patients with locally advanced cervical cancer (Keys *et al*, 1999; Morris *et al*, 1999; Rose *et al*, 1999; Whitney *et al*, 1999; Peters *et al*, 2000). This combined modality approach produced an absolute increase in 5-year survival of 12% compared with radiation therapy alone and has resulted in a dramatic change in the standard of care for this disease.

Although the general prognosis of patients with cervical cancer treated with radiation therapy has improved, how to best treat patients with residual disease after radiation therapy remains controversial. These patients have been considered to have an extremely poor prognosis (Moore *et al*, 2004; Long *et al*, 2005). The value of surgical treatment for such patients has been investigated, but in most of these studies, extended surgery, such as radical hysterectomy or pelvic exenteration, was performed, resulting in a high rate of severe treatment-related morbidity and decreased quality of life. For more than 30 years, we have performed simple abdominal hysterectomy in patients with residual disease identified 3 months after the completion of radiation therapy if the residual disease is central, without distant metastasis. The present study evaluated the treatment results of 35 cases who underwent so-called ‘adjuvant hysterectomy’ after radiation therapy.

## Methods

During the period 1971 through 1996, a total of 1590 new patients with primary invasive cervical carcinoma were treated by radiation therapy alone at the Cancer Institute Hospital, Tokyo, Japan. Disease stages were as follows: Ib, 197 patients; IIa, 29 patients; IIb, 620 patients; IIIa, 18 patients; and IIIb, 726 patients. Staging was performed according to the International Federation of Gynecology and Obstetrics criteria. A combination of EBRT and ICBT was used in all patients. The regimen for radiation therapy is described elsewhere (Ota *et al*, 2007). In brief, EBRT of a total dose of 50 Gy was delivered at 2 Gy per day, 5 days a week for 5 weeks. High-dose-rate remote after loading ICBT was also performed. A dose of 4 Gy was administered 2 or 3 times per week for a total of 40 Gy.

**Figure 1:**
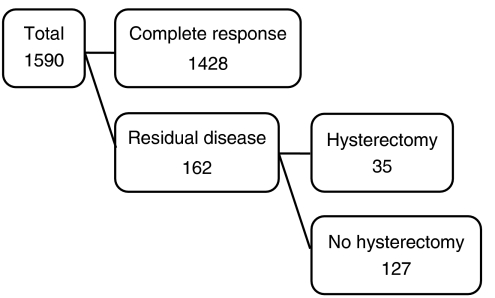
Incidence of local control in response to radiation therapy (complete response) and number of patients who underwent subsequent hysterectomy because of residual disease.

Three months after the completion of radiation therapy, the status of local control was investigated. Simple hysterectomy (class I hysterectomy) (Piver *et al*, 1974) was performed predominantly in incidence of patients with central residual disease within 2 months of discovery. The local control in response to radiation therapy and the number of patients who underwent subsequent hysterectomy are shown in Figure 1. Of the 1590 patients, 1428 experienced a complete response, as shown by cytologic and histologic assessment. Of 162 patients with persistent local disease, 35 underwent hysterectomy. The other 127 patients did not undergo surgery because of the presence of concomitant distant metastasis (*n*=26) or because of advanced age, poor medical condition, or refusal of surgery (*n*=101).

Follow-up examinations were performed every 3 months during the first 5 years after treatment and then at 6-month intervals for the next 5 years. All follow-up examinations included pelvic examination with cytologic assessment of the uterine cervix and tumour marker SCC antigen, and identification late complications. Every 6 months, we obtained CT scan of the abdomen and chest X-ray were performed. All patients were followed up for more than 10 years after radiation therapy. Our hospital is one of the few institutions permitted access to the family registry database. We consulted the district legal affairs bureau for survival information or the cause of death for each patient; none of the patients was lost to follow-up. Of the 1590 patients, 1323 (83.2%) were followed-up directly at the hospital, and 267 (16.8%) were followed-up through the district legal affairs bureau.

The survival data for each patient was calculated from the date therapy was started to the date of the latest follow-up examination. Survival curves were drawn according to the Kaplan–Meier method. The log-rank test was used for univariate analysis. *P* values less than 0.05 were considered statistically significant. With respect to radiation-related morbidity, late rectal and bladder complications and non-rectal gastrointestinal sequelae (small-bowel complications) were graded according to the *RTOG/EORTC* scoring system (Cox *et al*, 1995).

## Results

### Study population

The numbers of patients who underwent hysterectomy, listed by initial stage and cell type, are shown in Table 1. The mean age was 55.7 years (range, 36–74 years). Of the 35 patients, the number of patients with squamous cell carcinoma stage Ib, IIb, or IIIb were 3, 12, and 13, respectively, and the numbers with non-squamous cell carcinoma stage Ib, IIb, or IIIb disease were 1, 4, and 2.

### Hysterectomy

Details of the hysterectomy procedures are listed in Table 2. Type I hysterectomy was performed in 32 patients, and type III radical hysterectomy was performed in three patients; none of the patient underwent pelvic or para-aortic lymphadenectomy (Piver *et al*, 1974). The duration of surgery type for type I and type III hysterectomy was 95.6±34.1 and 158.0±32.9 min, respectively. Blood loss was 457.6±362.3 and 590.0±101.5 ml, respectively. Four patients showed residual disease after type I hysterectomy, and none of the patients showed residual disease after type III radical hysterectomy. Two of the four patients with residual disease, two underwent chemotherapy, and the other two underwent palliative treatment after hysterectomy. Thus, there were 31 patients with no residual disease after hysterectomy. However, recurrence developed in eight: local recurrence in four and distant recurrence in four.

### Survival

**Figure 2:**
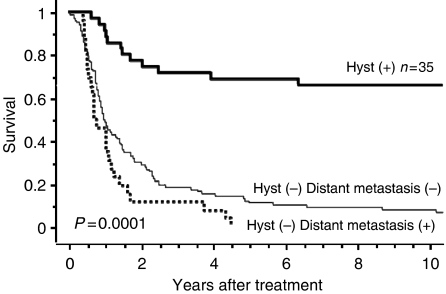
Disease-specific survival according to treatment methods and the presence or absence of distant metastasis for patients with cervical carcinoma. Hyst=Hysterectomy.

**Figure 3:**
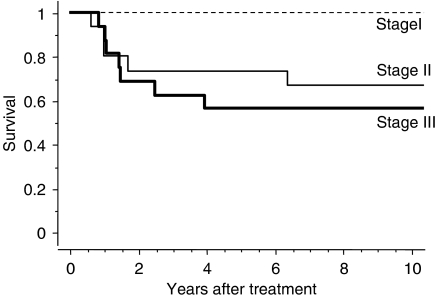
Disease-specific survival according to clinical stage for patients with cervical carcinoma.

**Figure 4:**
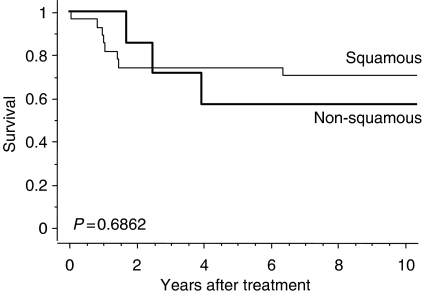
Disease-specific survival according to histologic type for patients with cervical carcinoma.

**Figure 5:**
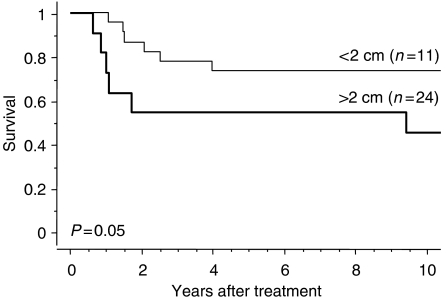
Disease-specific survival according to tumour size for patients with cervical carcinoma.

Disease-specific survival for the 35 patients who underwent hysterectomy is shown in Figure 2. The 5- and 10-year survival rates in this group were 68.6 and 65.7%, respectively. Clinical outcomes of patients who did not undergo hysterectomy, with or without distant metastasis, are also shown in Figure 2. Disease-specific 5-year survival rates for patients who did not undergo hysterectomy despite the absence of distant metastasis and those with concomitant distant metastasis were 14.5 and 0%, respectively. Significant differences were noted between the three groups (*P*=0.0001). Disease-specific survival curves are shown according to cancer stage in Figure 3. The cumulative 5-year survival rates for initial stages Ib, IIb, and IIIb disease were 100, 73.3, and 56.3%, respectively, and 10-year survival rates for initial stages Ib, IIb, and IIIb disease were 100, 66.7, and 56.3%, respectively. Disease-specific survival curves are shown according to histologic type in Figure 4. The 5- and 10-year survival rates for patients with squamous cell carcinoma were 74.1 and 70.4%, respectively, and those for patients with non-squamous cell carcinoma were both 57.1%. No difference was observed in survival between patients with squamous cell carcinoma and those with non-squamous cell carcinoma (*P*=0.6862). Disease-specific survival curves are shown according to tumour size in Figure 5. The size of the persistent tumour at the time of hysterectomy was important with respect to survival. The 5- and 10-year survival rates for patients with lesions less than 2 cm in diameter were both 75.0% and those for patients with larger lesions were 54.4 and 45.5%, respectively. There was a trend for better survival of smaller tumours compared with larger tumours, but this difference was not statistically significant (*P*=0.053).

### Postoperative complications

One patient who suffered postoperative small-bowel obstruction was treated conservatively, and was discharged 40 days after surgery. Six patients (17.1%) experienced pelvic or urinary tract infection, but none required surgical intervention. No treatment-related deaths occurred.

### Late complications

Late complications are listed in Table 3. According to the *RTOG/EORTC* scoring system, grade III or IV late complications involving the rectum, small-bowel, or urinary tract were observed in five (14.3%) cases, three were stage II and two were stage III. The incidences of grade III and grade IV rectal complications were 0 and 2.9% (one patient), respectively. None of the patients experienced grade III or grade IV small-bowel complications. The incidences of grade III and grade IV urinary tract complications were 2.9% (one patient) and 5.7% (two patients), respectively. One patient (stage III disease) required reconstruction of both the urinary tract and lower gastrointestinal tract.

## Discussion

There is no standard treatment for residual cervical carcinoma in cases in which radiation therapy is insufficient. Patients with residual disease often undergo chemotherapy, with only minor palliation and without any significant improvement in survival (Moore *et al*, 2004; Long *et al*, 2005). Other treatments include re-irradiation or interstitial irradiation offering local control rates of 64–92% along with a 5-year survival rate of 4–44% for recurrent cervical cancer. However, a high rate of severe complications has been reported in both the urinary and lower gastrointestinal tracts (Russell *et al*, 1987; Sommers *et al*, 1989; Xiang-E *et al*, 1998).

With respect to surgical treatment for residual disease after radiation therapy, the most effective method is probably pelvic exenteration (Stanhope *et al*, 1990). Total pelvic exenteration offers a 5-year survival rate of 23–50%, but it requires alterations of both the urinary and lower gastrointestinal tracts, and a high rate of severe postoperative complications, such as infection, injury of the urinary and lower gastrointestinal tracts, and small-bowel obstruction, occur, in addition to a 4–14% surgery-related mortality rate (Rutledge *et al*, 1977; Morley *et al*, 1989; Shingleton *et al*, 1989; Matthews *et al*, 1992; Moutardier *et al*, 2004).

The utility of radical hysterectomy, a more conservative procedure, has been reported, but it also has a high rate of complications. Coleman *et al* (1994) evaluated the utility of radical hysterectomy (chiefly type III hysterectomy) including pelvic lymphadenectomy in 50 patients with persistent or recurrent cervical cancer after primary radiation therapy. The 5- and 10-year survival rates for all cases was 72 and 60%, but severe postoperative complications (grade III or higher) occurred in 42% of the patients, along with one postoperative death because of sepsis. The most common site of injury was the urinary tract, with 14 patients (28%) developing vesicovaginal fistula, 11 (22%) developing ureteral injuries, and 10 (20%) developing severe long-term bladder dysfunction. Maneo *et al* (1999) evaluated the utility of type III radical hysterectomy including pelvic lymphadenectomy in 34 patients with persistent or recurrent cervical cancer after primary radiation therapy. The 5-year survival rate for all cases was 49%. No treatment-related deaths or early postoperative complications occurred, but 18 major complications occurred in 15 (44%) of the patients. Rutledge *et al* (1994) studied 47 patients with persistent or recurrent cervical cancer after primary radiation therapy and reported that radical hysterectomy resulted in major complications in 20 (42.4%) of the patients, including two treatment-related deaths. These results suggest that radical hysterectomy including pelvic lymphadenectomy can be an alternative to exenteration, but the high incidence of treatment-related morbidity remains a major issue.

The necessity of lymphadenectomy in recurrent or persistent disease should be addressed. Coleman *et al* (1994) reported that all five patients with positive nodes died of cancer, whereas 14 of 34 patients (41.2%) with negative nodes died of cancer (*P*=0.02). Maneo *et al*, (1999) identified six node-positive patients in their series, and five experienced recurrence, whereas half of 28 node-negative patients experienced recurrence. Thus, it is likely that pelvic lymphadenectomy resulted in little, if any, improvement of survival in residual disease after radiation therapy. Results of the present study, along with these previous studies, suggest that abdominal simple hysterectomy without pelvic lymphadenectomy (class II hysterectomy) may be sufficient in eliminating residual disease after radiation therapy.

The value of adjuvant hysterectomy in the treatment of cervical cancer after radiation therapy has been investigated; most of these studies suggest a decreased incidence of local relapse but no benefit in progression-free survival (Perez *et al*, 1987; Keys *et al*, 2003). Keys *et al* (2003) reported in a Gynecologic Oncology Group trial that performing adjuvant hysterectomy in every case of cervical cancer after radiation therapy is of little value in improving survival, although no significant increase in treatment-related morbidity is observed. In addition, Whitney *et al* (1999) reported that 7 of 30 (23.3%) stage IB patients with residual disease showed recurrence, whereas only 1 of 50 patients (2%) showed recurrence in the absence of evidence of persistent residual disease. Gallion *et al* (1985) reported similar results, with 5 of 14 (35.7%) residual disease showing recurrence compared to 2 of 29 (6.9%) with no residual disease. Thus, our belief is that adjuvant hysterectomy should be performed only in cases of residual disease of the cervix and that surgical treatment is unnecessary in cases with no residual disease.

It is noteworthy that adjuvant hysterectomy for residual disease after radiation therapy can be applied for patients with non-squamous cell carcinoma or stage III disease. Previous reports suggest that adenocarcinoma of the cervix has a poor prognosis (Eifel *et al*, 1990). Nakano *et al* (1995) report resulted of 58 patients with adenocarcinoma of the cervix treated with radiation. The local control rates in stage III and stage IV cases were 56 and 27%, respectively, and 5-year survival rates were 32.3 and 9.1%. These findings suggest that patients with adenocarcinoma who have residual disease as a failure of radiation therapy often have a poor prognosis in the absence of appropriate treatment for the residual disease. Gerdin *et al* (1994) performed radiation therapy and adjuvant hysterectomy in all of their cases, and reported successful results in the treatment of non-squamous cervical cancer and that the histologic tumour type is not a significant prognostic factor. In the present study, we also found no significant difference in the survival between patients with squamous cell carcinoma and those with non-squamous cell carcinoma.

Residual stage III disease is seldom treated surgically. The present study showed 5- and 10-year survival rates for patients with stage III disease of 56.3%, with acceptable treatment-related morbidity, suggesting that adjuvant hysterectomy may be of value in the treatment of advanced cervical cancer.

Previous studies have reported a correlation between tumour size at surgery and survival rate. Coleman *et al* (1994) reported that tumour size at surgery is significantly associated with survival. The 5-year survival rate for the 12 of 44 patients (27.3%) with lesion diameter less than 2 cm was 90% compared with 64% in patients with larger lesions (*P*<0.01). Maneo *et al* (1999) also reported that smaller tumour size (<4 cm) in recurrent disease is predictive of survival. In the present study, there was a trend for better survival smaller-sized tumours (<2 cm) compared with those with larger-sized tumours.

In conclusion, adjuvant hysterectomy is a viable option when applied to patients in whom an incomplete response to radiation leaves residual cervical carcinoma. This treatment is also effective in patients with stage III disease and in those with non-squamous cell carcinoma.

## Figures and Tables

**Table 1:** Case of cervical carcinoma treated with hysterectomy after radiation therapy

**Clinical stage**	**Number of patients**
*Squamous cell carcinoma*	
Stage Ib	3
Stage IIb	12
Stage IIIb	13
	
*Non-squamous cell carcinoma*	
Stage Ib	1
Stage IIb	4
Stage IIIb	2
	
Total	35

**Table 2:** Hysterectomy

*Type I hysterectomy (n=32)*	
Duration of surgery	95.6±34.1 min
Blood loss	457.6±362.3 ml
Residual disease	4 patients
	
*Type III radical hysterectomy (n=3)*	
Duration of surgery	158.0±32.9 min
Blood loss	590.0±101.5 ml
Residual disease	0 patients

**Table 3:** Grades of late complications according to site

	**Grade III (%)**	**Grade IV (%)**	**Grade V (fatal)**
Rectum	—	1 (2.9)	—
Small bowel	—	—	—
Bladder	1 (2.9)	2 (5.7)	—
Combined	—	1 (2.9)	—
			
Total	1 (2.9)	4 (11.4)	—

Total: Five cases (14.3%).
